# Supplementary material for: Statistical Triage Model for Feline Infectious Diseases in a Veterinary Isolation Unit: The Case of Feline Immunodeficiency and Leukemia Viruses
Source: Vet Sci. 2025 Sep 17;12(9):902. doi: 10.3390/vetsci12090902 (PMC12474253; doi:10.3390/vetsci12090902)
Supplement: Supplementary file 1 [file vetsci-12-00902-s001.zip › Table_S2.pdf]

Table S2- Performance metrics of FIV and FeLV logistic regression models

Metric	FIV – ROC01	FIV – Youden	FeLV – ROC01	FeLV – Youden
Optimal cutoff	0.22	0.19	0.22	0.16
Sensitivity (%)	65.7	78.4	65.1	78.6
Specificity (%)	63.5	52.2	60.5	49.8
Positive Predictive Value (PPV, %)	32.4	30.3	29.2	28.1
Negative Predictive Value (NPV, %)	87.4	90.1	87.4	90.3
