# Supplementary material for: Statistical Triage Model for Feline Infectious Diseases in a Veterinary Isolation Unit: The Case of Feline Immunodeficiency and Leukemia Viruses
Source: Vet Sci. 2025 Sep 17;12(9):902. doi: 10.3390/vetsci12090902 (PMC12474253; doi:10.3390/vetsci12090902)
Supplement: Supplementary file 1 [file vetsci-12-00902-s001.zip › Table_S1..pdf]

Table S1. Association between FIV and FeLV seropositivity

	FeLV +	FeLV –
FIV +	27	134
FIV –	138	568

$\chi^2 = 0.489$, $p = 0.485$; Fisher's $p = 0.504$
