# Supplementary figures and images for: Statistical Triage Model for Feline Infectious Diseases in a Veterinary Isolation Unit: The Case of Feline Immunodeficiency and Leukemia Viruses
Source: Vet Sci. 2025 Sep 17;12(9):902. doi: 10.3390/vetsci12090902 (PMC12474253; doi:10.3390/vetsci12090902)

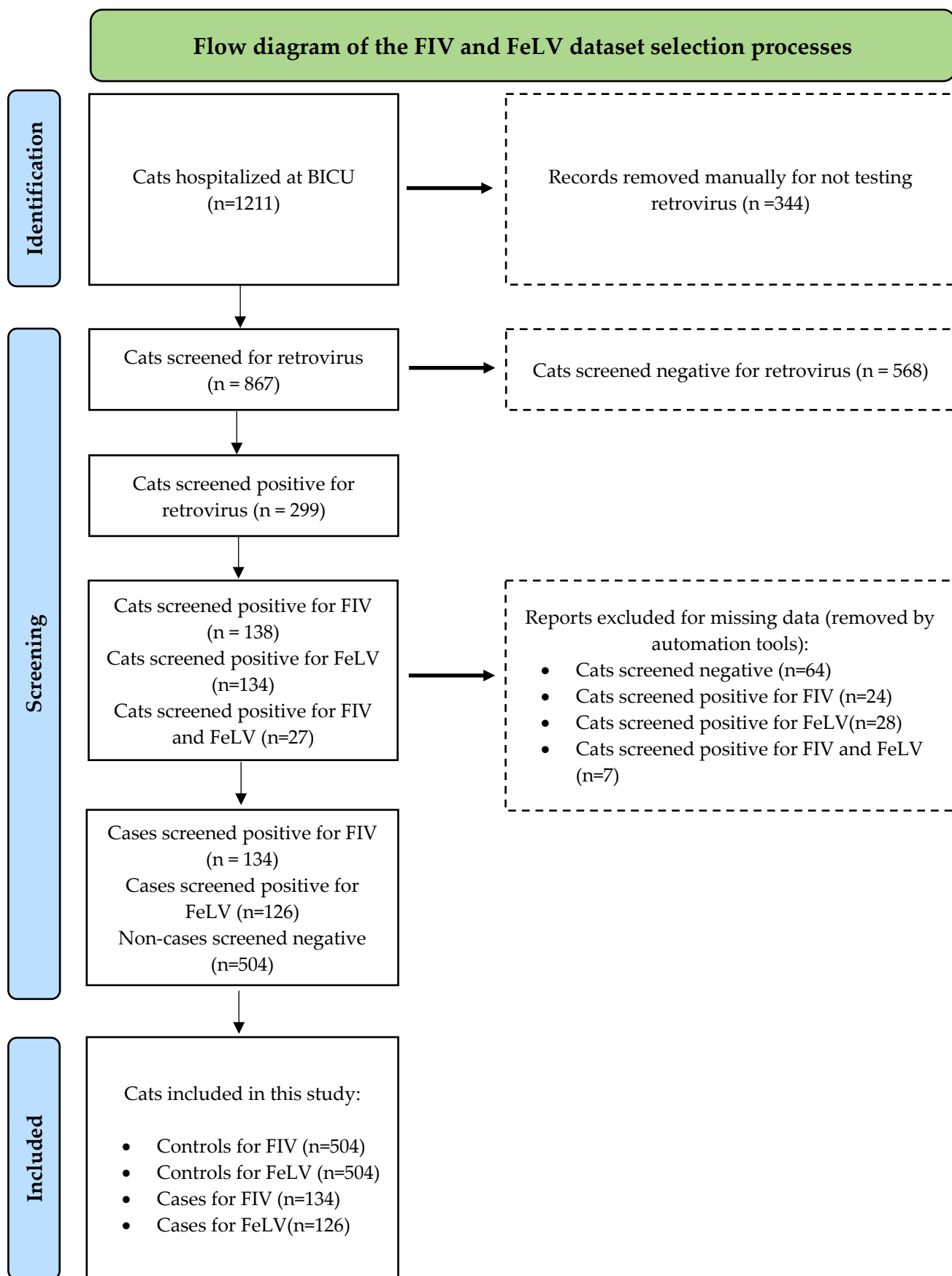

**Figure S1.** Flow diagram of the FIV and FeLV dataset selection processes

Supplement: Supplementary file 1 [file vetsci-12-00902-s001.zip › Suplementary Figure S1.pdf]
